# The prevalence of malnutrition based on anthropometry among primary schoolchildren in Binh Dinh province, Vietnam in 2016

**DOI:** 10.3934/publichealth.2018.3.203

**Published:** 2018-06-26

**Authors:** Truong Quang Dat, Le Nguyen Huong Giang, Nguyen Thi Tuong Loan, Vo Van Toan

**Affiliations:** 1 Binh Dinh Medical College, Vietnam; 2 Quy Nhon University, Vietnam

**Keywords:** thinness, stunting, underweight, obesity, primary schoolchildren, Binh Dinh, Vietnam

## Abstract

**Objective:**

The study was conducted to determine the prevalence of malnutrition based on anthropometry among primary schoolchildren in Binh Dinh province, Vietnam.

**Material and Methods:**

This was a school-based cross-sectional survey using random sample technique with multistage process. Variables in malnutrition were classifed as thinness, stunting, underweight, overweight, and obesity based on z-scores according to the World Health Organization (WHO) (2007). Anthropometric measurements were taken according to WHO's standard procedures. The Chi-square test was used to compare prevalences and the Chi-square test for trend was employed to assess the trend of the prevalence of malnutrition forms by age.

**Results:**

6,514 pupils from 6 to 10 years old including 3,298 males and 3,216 females were observed. The prevalence of thinness, stunting, underweight, overweight and obesity among schoolchildren accounted for 11.19%, 6.16%, 10.79%, and 30.1%, respectively. The prevalence of underweight and that of overweight-obesity of all pupils at the age of 6 were 13.1% and 32.11%, respectively, and tended to decrease to age 10 (*p* < 0.01). The prevalence of thin and stunted pupils had little sign of change over ages (*p* > 0.05). There was a statistically significant difference in the prevalence of malnutrition in three areas of Binh Dinh (*p* < 0.05), in which the highest prevalence of undernutrition was in mountainous area and midland, and the highest prevalence of overweight-obesity was in urban areas.

**Conclusion:**

The prevalence of malnutrition of primary schoolchildren in Binh Dinh was relatively high, in which the prevalence of overweight-obesity was rather high in urban areas and the prevalence of undernutrition was pretty high in mountainous area and midland. This study has characterized an important public health challenge, highlighting the need for attention to potential interventions.

## 1. Introduction

Malnutrition refers to deficiencies, excesses, or imbalances in a person's intake of energy and/or nutrients. The term malnutrition addresses 3 broad groups of conditions: undernutrition, which includes wasting (low weight-for-height), stunting (low height-for-age) and underweight (low weight-for-age); micronutrient-related malnutrition, which includes micronutrient deficiencies (a lack of important vitamins and minerals) or micronutrient excess; and overweight, obesity and diet-related noncommunicable diseases (such as heart disease, stroke, diabetes and some cancers) [1]. According to WHO's Classification of nutrition conditions based on anthropometry in children over 5 years old and adolescents, malnutrition generally refers to undernutrition and overnutrition, in which undernutrition includes thinness (low Body Mass Index-for-age (BMI-for-age)), stunting (low height-for-age), underweight (low weight-for-age); overnutrition includes overweight and obesity (high BMI-for-age) [2]. Childhood malnutrition remains a major public health problem in developing countries and a major contributor to global disease burden [3]. It is estimated that 159 million pre-school children were stunted while 41 million pre-school children became obesity in 2014. Globally, pre-school childhood stunting decreased from 39.6% in 1990 to 23.8% in 2014. Also in this year, overweight or obesity increased in all regions, from 4.8% in 1990 to 6.1% [4], especially the prevalence of pre-school children with overweight and obesity in low-income and middle-income countries increased dramatically [5],[6]. These increasing trends are conjectured to continue, with 60 million children under 5 expected to be overweight or obese in 2020 [7].

In Vietnam, the underweight prevalence of children under 5 has gained its steady downward trend over the past 5 years, from 17.5% in 2010 to 14.1% in 2015, achieving the planned targets for 2015 (below 15%) and is expected to continue to decline in the coming years. The stunting malnutrition prevalence has also declined during this period, from 29.3% in 2010 to 24.2% in 2015. This represented a reduction of more than 60% in the prevalence of underweight malnutrition in children as compared to 1990. While underweight has dropped rapidly, the obesity prevalence in children under age 5 is growing rapidly. In 2010 the prevalence was 4.8%, six times higher than that in children in 2000 [7]–[9]. In addition, the stunting prevalence had also declined during this period, from 29.3% in 2010 to 24.2% in 2015, but still high.

These states of malnutrition of children under age 5 are likely to continue to have a long-term impact on their health including nutritional status in older age groups, especially in primary school age. Malnutrition affects all aspects of children's life; its impacts are not limited to physical health but extend to mental, social, and spiritual well-being as well as school achievement [10]–[12]. Primary schoolchildren, from 6 to 10 years old, are in the period for accumulation of nutrients needed to prepare for the next stage of their puberty, and this phase also involves in learning at school and adapting to the social environment. Moreover, the progression of puberty is affected by nutrition. Good nutrition during the period of time helps children improve their health and resistance to disease, create good growth momentum in the next stage and develop appropriate height when they mature. Some evidence suggests that obesity can accelerate the onset of puberty in girls and may delay the onset of puberty in boys [13]. In developing countries, the prevalence of malnutrition forms among schoolchildren varies from country to country [14]. In Vietnam, a number of studies showed that the prevalence of overweight and obesity of primary schoolchildren used to be significantly high, especially in big cities [15]–[16]. Besides, the prevalence of stunting, thinness and underweight among schoolchildren remained high and did not seem to be better in several areas [17].

Located in the central coast of Vietnam, Binh Dinh has the population of 1,501,800 people in which there are about 122,500 primary schoolchildren all over the province [18]. Also in Vietnam, particularly in the study area, there is scarcity of information on malnutrition among primary schoolchildren. Since most previous studies were conducted on prevalence of malnutrition merely among under-5-year-old children in Binh Dinh, we surmise that identifying malnutrition indicators among schoolchildren in the province surely is an essential step to set a sustainable and effective nutritional intervention in the study area. Thus, the present study was designed to identify the prevalence of malnutrition forms based on anthropometry among primary schoolchildren in Binh Dinh Province in 2016.

## 2. Materials and methods

### 2.1. Study design

This is a cross-sectional study based on schools. It was conducted in three socio-economic areas of Binh Dinh Province including: (1) urban, (2) coastal plain, and (3) mountainous area and midland in which the last is the most disadvantageous socio-economic region according to the socio-economic classification of the administrative units directly under the province. The last has the highest percentage of poor households and the lowest per capita incomes [18]. The study was carried out on pupils from 6 to 10 years old in September 2016. Pupils with physical deformities, recent history of acute (diarrhea, measles, mumps, chicken pox, etc.) or chronic (asthma, diabetes, cardio-vascular disease, epilepsy, tuberculosis, dermatitis, intestinal problems, etc.) diseases were excluded from the study.

### 2.2. Sampling

Sample size was calculated using the following formula: ${\text{n}}_{1}={\text{Z}}_{1-\text{α}/2}^{2}\frac{\text{p }(1-\text{p})}{{\text{d}}^{2}}$; Where: n_1_ is the smallest sample size to be achieved for each grade for one area; p is the expected prevalence: 23.4% [19]; d is the absolute error: 5%; Z(1-α/2) is Z statistic for a level of confidence of 95%. Sample size was adjusted to take account of the stratification into five age groups from 3 socio-economic areas: n_2_ = n_1_ × 5 × 3 × 1.5 = 5,790. Final sample size (n = 6,100) was determined by adjusting for expected non-respone (5%). In this study, a total of 6,514 children (with 3,298 (50.63%) males and 3,216 (49.37%) females) ranging from 6 years old up to 10 years old were surveyed in 3 socio-economic regions of Binh Dinh province.

Sampling method: The random sample technique with multistage process was adopted to select the study population. There are 11 administrative units under Binh Dinh Province divided into 3 socio-economic areas: urban area (including Quy Nhon city and An Nhon town), coastal plain area (including districts of Tuy Phuoc, Phu Cat, Phu My and Hoai Nhon), and mountainous area and midland (including the rest of the province). Stage 1: Randomly selected 1 unit among the urban area, 2 districts among 4 coastal plain districts and 2 disctricts among mountainous districts and midland. Stage 2: There were 106 primary schools in the selected units. Based on the number of pupils in each school, we decided to choose 18 schools to ensure the required sample sizes, in which the number of schools selected for the study in the urban area, the coastal plain area and the mountainous area and midland was 4, 6 and 8 respectively. Final stage: All pupils of the selected schools would be in the research sample.

### 2.3. Data collection

Weight and height measurements were carried out according to the following standard operating procedures. Weight was measured to the nearest 0.1 kg in a standard weighing scale (model TANITA made in Japan). Children were asked to stand still on the centre of the scale with minimal clothing and without footwear and kept their head up and face forward, arms hanging freely by the sides of the body, with palms facing the thigh. Height was measured to the nearest 0.1 cm with nonstretchable tape which was fixed to a vertical smooth wall and the participants were asked to stand erect without footwear on a firm surface with his/her back against the wall, feet parallel, and hands hanging by the sides. Each measurement was done twice and the average of the two readings was recorded.

### 2.4. Key variables

Variables in malnutrition were classifed using the following categories: thinness, stunting, underweight, overweight and obesity. Criteria for assessing malnutrition based on anthropometry were based on z-scores according to WHO (2007) [2].

Thinness was defined as low BMI-for-age. Children with z scores (BMIZ) under-2SD to-3SD were considered as moderate thinness and those with BMIZ under -3SD were considered as severe thinness. Stunting was defined as low height-for-age. Children with z scores (HAZ) under-2SD to-3SD were considered as stunting and those with HAZ under -3SD were considered as severe stunting. Underweight was defined as low weight-for-age. Children with z-scores (WAZ) under-2SD to-3SD were considered as underweight and those with WAZ under-3SD were considered severe underweight. Overweight and obesity were defined as BMI-for-age. Children with BMIZ over +1SD to +2SD were considered as overweight and those with BMIZ over +2SD were considered obesity.

### 2.5. Data processing

After cleaning, data were entered into Epi Data 3.1 software and transferred to Stata 10.0 software for data analysis. Absolute values and percentages were used to describe categorical variables. The Chi-square test for trend [20] was used to assess the trend of the prevalence of malnutrition forms by age. Then the Chi-square test was utilized to compare prevalence in groups. A *p*-value< 0.05 was considered statistically significant.

### 2.6. Ethical considerations

The study was approved by the Ethical Commitee of Hue University. The research was allowed by Education and Training Committee Division in the selected districts and city, and the primary schools at the research sites. All participants voluntarily participated. The data collected were kept confidential. Cases of malnutrition were advised on nutrition. The study results were to be disseminated to relevant stakeholders in order to inform policies and interventions to improve the nutritional status of schoolchildren and paved the way for future studies.

## 3. Results

The data set included the records of 6,514 children (with 3,298 (50.63%) males and 3,216 (49.37%) females) ranging from 6 years old up to 10 years old ranging from 6 years old up to 10 years old. Gender distribution for each area was similar. The size of the samples varied from 2,139 for the coastal plain area; 2,335 for the urban area to 3,216 for the mountainous area and midland. Similarly, sample sizes by age group from 6 to 10 were 1,305 (20.03%); 1303 (20%); 1,279 (19.63%); 1,275 (19.57%) and 1,352 (20.76%) respectively. Age distribution in the sample was similar between age groups.

As shown in Table 1, children with thinness, stunting, underweight, overweight and obesity were 11.19%, 6.16%, 10.79% and 30.1%, respectively. There was a variation for the prevalence of stunting, ranging from 5.22% in males to 7.12% in females (*p* = 0.001). The total of overweight and obesity of males (33.78%) was higher than that of females (26.34%) with *p*-value of 0.00. However, differences in the prevalence of thinness and underweight in both genders were not statistically significant.

**Table 1.** Prevalence of malnutrition forms among primary school pupils based on BMI, height and weight by sex

Malnutrition forms		Total (n = 6,514)	Males (n_m_ = 3,298)	Females (n_f_ = 3,216)	*p*
		No (%)	No (%)	No (%)	
Severe thinness	< -3SD	173 (2.66)	99 (3.00)	74 (2.30)	0.08
Moderate thinness	< -2SD to -3SD	556 (8.54)	278 (8.43)	278 (8.64)	0.76
*Total of thinness*	*< -2SD*	*729 (11.19)*	*377 (11.43)*	*352 (10.95)*	*0.53*
Severe stunting	< -3SD	45 (0.69)	22 (0.67)	23 (0.72)	0.82
Moderate stunting	< -2SD to -3SD	356 (5.47)	150 (4.55)	206 (6.41)	0.001
*Total of stunting*	*< -2SD*	*401(6.16)*	*172 (5.22)*	*229 (7.12)*	*0.001*
Severe underweight	< -3SD	142 (2.18)	83 (2.52)	59 (1.83)	0.06
Moderate underweight	< -2SD to -3SD	561 (8.61)	275 (8.34)	286 (8.89)	0.43
*Total of underweight*	*< -2SD*	*703 (10.79)*	*358 (10.86)*	*345(10.73)*	*0.88*
Overweight	> +1SD to +2SD	1,081(16.60)	497(15.07)	584 (18.16)	0.00
Obesity	> +2SD	880 (13.51)	617(18.71)	263 (8.18)	0.00
*Total of overweight and obesity*	*> +1SD*	*1,961(30.10)*	*1,114 (33.78)*	*874(26,34)*	*0.00*

Note: The *p* is probability value obtained in the test for comparison of malnutrition forms between genders.

Table 2 showed the prevalence of malnutrition forms for each gender by age. The prevalence of thinness and stunting among all pupils varied across ages (from 6 to 10 years old) but not statistically significant (*p* = 0.29 and 0.3 respectively). The prevalence of underweight and overweight-obesity among all pupils tended to decline as age increased (*p* = 0.00 and 0.05 respectively). There were some differences in trends in malnutrition among boys and girls. There was a tendency for the prevalence of stunting to decrease in boys, starting in age 7 while this tendency was opposite in girls. The prevalence of overweight and obesity tended to little decrease from 6 to 10 years old in boys but significantly decrease in girls (*p* = 0.001) in the same age group.

Results of prevalence of malnutrition forms among primary school pupils by socio-economic areas were presented in Table 3. There were statistically significant differences in the abnormal nutritional status in the three areas in which the undernutrition prevalence was the highest in the mountainous area and midland; overweight-obesity was the highest in the urban area. The prevalence of thinness among pupils in the urban area, the coastal plain area, and the mountainous area and midland was 2.23%, 10.99% and 21.67% respectively (*p* = 0.00) while the prevalence of overweight and obesity among pupils in those three areas was 59.07%, 18.42% and 11.47% respectively (*p* = 0.00).

**Table 2.** Prevalence of malnutrition forms among primary school pupils based on BMI, height and weight by age

Malnutrition forms	6 years (n_6_ = 1,305)	7 years (n_7_ = 1,303)	8 years (n_8_ = 1,279)	9 years (n_9_ = 1,275)	10 years (n_10_ = 1,352)	*p*
	No (%)	No (%)	No (%)	No (%)	No (%)	
*Boys*						
Thinness	81 (12.37)	91 (13.09)	76 (11.97)	62 (9.58)	67 (10.06)	0.04
Stunting	46 (7.02)	40 (5.76)	33 (5.20)	18 (2.78)	35 (5.26)	0.02
Underweight	87 (13.28)	79 (11.37)	97 (15.28)	51 (7.88)	44 (6.61)	0.00
Overweight and obesity	224 (34.20)	246 (35.40)	201 (31.65)	223 (34.47)	220 (33.03)	0.56
*Girls*						
Thinness	73 (11.23)	69 (11.35)	59 (9.16)	61 (9.71)	90 (13.12)	0.53
Stunting	37 (5.69)	42 (6.91)	27 (4.19)	53 (8.44)	70 (10.20)	0.001
Underweight	84 (11.92)	58 (9.54)	47 (7.30)	89 (14.17)	67 (9.77)	0.61
Overweight and obesity	195 (30.00)	145 (23.85)	216 (33.54)	139 (22.13)	152 (22.16)	0.001
*All pupils*						
Thinness	154 (11.80)	160 (12.28)	135 (10.56)	123 (9.65)	157 (11.61)	0.29
Stunting	83 (6.36)	82 (6.29)	60 (4.69)	71 (5.57)	105 (7.77)	0.30
Underweight	171 (13.10)	137 (10.51)	144 (11.26)	140 (10.98)	111 (8.21)	0.00
Overweight and obesity	419 (32.11)	391 (30.01)	417 (32.60)	362 (28.39)	372 (27.51)	0.007

Note: The *p* is probability value obtained in the nonparametric test for trends of the prevalence of malnutrition forms across primary school ages.

**Table 3.** Prevalence of malnutrition forms among primary school pupils by socio-economic areas

Malnutrition forms	The urban area (n_u_ = 2,335)	The coastal plains area (n_c_ = 2,139)	The mountainous area and midland (n_m_= 3,216)	*p*
	No (%)	No (%)	No (%)	
Severe thinness	17 (0.73)	44 (2.06)	112 (5.49)	0.00
Moderate thinness	35 (1.50)	191 (8.93)	330 (16.18)	0.00
*Total of thinness*	*52 (2.23)*	*235 (10.99)*	*442 (21.67)*	*0.00*
Severe stunting	3 (0.13)	6 (0.28)	36 (1.76)	0.00
Moderate stunting	55 (2.36)	82 (3.83)	219 (10.74)	0.00
*Total of stunting*	*58 (2.48)*	*88 (4.11)*	*255 (12.50)*	0.00
Severe underweight	13 (0.56)	22 (1.03)	107 (5.25)	0.00
Moderate underweight	36 (1.54)	160 (7.48)	365 (17.89)	0.00
*Total of underweight*	*49 (2.10)*	*182 (8.51)*	*472 (23.14)*	*0.00*
Overweight	635 (27.19)	284 (13.28)	162 (7.94)	0.00
Obesity	698 (29.89)	110 (5.14)	72 (3.53)	0.00
*Total of overweight and obesity*	*1,333 (57.09)*	*394 (18.42)*	*234 (11.47)*	*0.00*

Note: The *p* is probability value obtained in the test for comparison of malnutrition forms between the three study areas.

**Figure 1.**
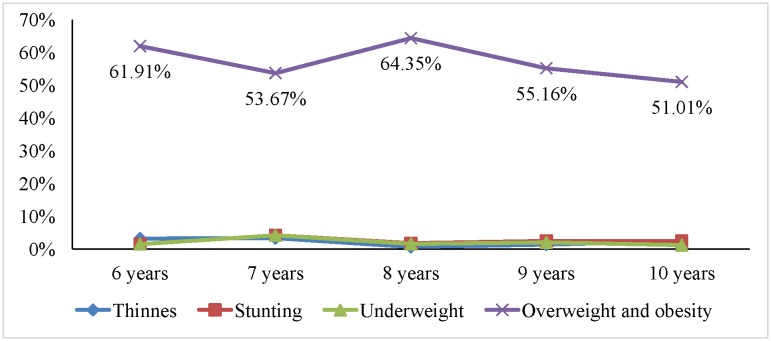
Trends of malnutrition forms among primary school pupils in the urban area based on BMI, height and weight by age. Note: The *p* is probability value obtained in the nonparametric test for trends of the adverse forms of nutrition across primary school ages. *p* value < 0.01 was for overweight-obesity.

**Figure 2.**
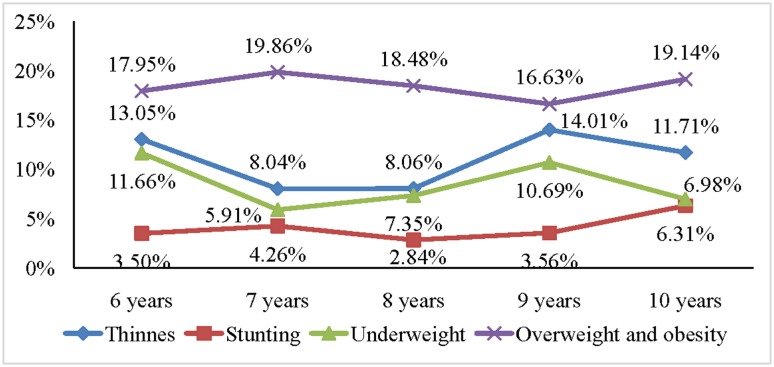
Trends of malnutrition forms among primary school pupils in the coastal plain area based on BMI, height and weight by age. Note: The *p* is probability value obtained in the nonparametric test for trends of the adverse forms of nutrition across primary school ages. *p* value > 0.05 in all adverse types of nutritional status.

**Figure 3.**
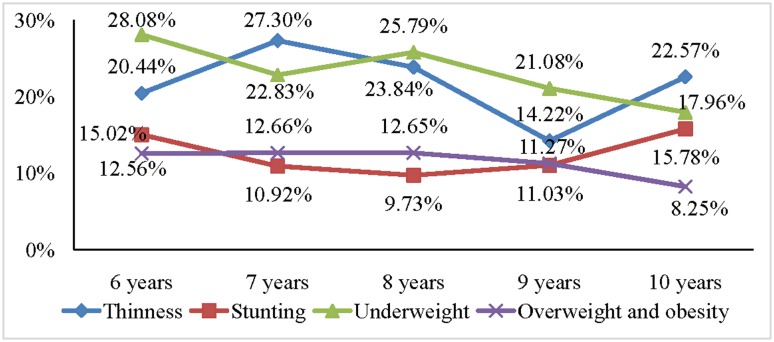
Trends of malnutrition forms among primary school pupils in the mountainous area and midland based on BMI, height and weight by age. Note: The *p* is probability value obtained in the nonparametric test for trends of the adverse forms of nutrition across primary school ages. *p* value < 0.05 was for underweight and overweight-obesity.

Figure 1, Figure 2 and Figure 3, respectively, illustrated trends of malnutrition forms among primary school pupils in the three socio-economic regions of the province based on BMI, height and weight by age. In the urban area, the prevalence of malnutrition forms such as thinness, stunting among pupils varied across ages (from 6 to 10 years old) but not statistically significant. The prevalence of overweight-obesity among pupils in this area tended to decline as age increased (*p* = 0.004). In the coastal plain area, malnutrition forms such as thinness, stunting, underweight, overweight and obesity varied over age, but those variations were not statistically significant. In the mountainous area and midland, the prevalence of malnutrition forms such as thinness, stunting among pupils varied across ages (from 6 to 10 years old) but not statistically significant. However, the prevalence of underweight and overweight-obesity among pupils in those areas tended to decline as age increased (*p* = 0.04).

## 4. Discussion

The present study showed that almost 10% of all children attending primary schools in Binh Dinh province suffered from at least one type of undernutrition in which thinness was 11.19%, higher than stunting and underweight. Meanwhile, 16.6% and 13.51% of children were overweight and obese respectively. In this study, the overall prevalence of stunting was 6.16% which is lower than that in studies in Eastern Ethiopia 8.9% [21], in Burkina Faso 8.8% [22], in Pakistan 10% [23], in Baghdad Iraq 18.7% [24] and in Tien Hai-Thai Binh-Vietnam 28.6% [19]. Yet, it is higher than that in Ho Chi Minh city-Vietnam 3.5% [16] and similar to that in Van Giang district, Hung Yen province-Vietnam 5.9% [25]. The prevalence of thinness in our study is similar to that in Tien Hai-Thai Binh [19], but the prevalence of underweight is much higher than that in Van Giang [25]. These differences may be due to the differences in socio-economic, culture, feeding habits, environmental factors, and public service utilization of the community in the study sites. The prevalence of overweight-obesity among primary school children in Binh Dinh for all ages was 30.1% which is much higher than that in Tien Hai district 1.6% [19] but much lower than that in Ha Noi, Tay Ninh-Vietnam in which the prevalence overweight-obesity of children at the same age group is 38.2% [15] and 34% [26] respectively. Obviously, rapid economic development and urbanization in Binh Dinh in recent years have caused a rise to the nutrition transition, where energy-dense diets replace traditional diets and sedentary lifestyles prevail. As a result of that, the dramatic increase in the prevalence of overweight and obesity in Binh Dinh like other developing countries [27] and it has also been reported in several recent studies in Vietnam [16],[28].

The results showed that the prevalence of thinness and underweight was similar among boys and girls, but there was a statistically significant difference in the both sexes of the prevalence of stunting and overweight-obesity. The prevalence of stunting was significantly higher among girls (7.13%) than boys (5.22%) with *p*-value of 0.001 while the prevalence of overweight and obesity was significantly higher among boys (33.78%) than girls (26.34%) with *p*-value of 0.00. Such findings were also found in the survey on nutritional status of primary school pupils in District 1 in 2003 and District 10 in Ho Chi Minh city for the 2008–2009 school year [29] and in some other studies in Vietnam [15],[30]–[32]. As a result, to clarify this issue, more research should be done to find out the reasons in order to have appropriate interventions in highly risk populations.

This study also showed the tendency of the prevalence of malnutrition forms among primary school pupils in Binh Dinh. At the age of 6, the prevalence of thinness and stunting among pupils were 11.8% and 6.36%, respectively; however, these two forms of malnutrition were little changed over the age ranging from 6 to 10 years old (*p* > 0.05). For a more detailed analysis, we found that the prevalence of boys with thinness, stunting and underweight at the age of 6 tended to decrease from 6 to 10 years old in which the prevalence was 10.06%, 5.26% and 6.61% respectively at the age of 10 (*p* < 0.05) while the prevalence of girls with stunting tended to increase from 6 to 10 years old (*p* = 0.001). These results showed that there was a difference in undernutrition trends in the two sexes. It was possibly due to the fact that nutritional interventions for children under 5 or primary schoolchildren were ineffective or may be less noticeable. The prevalence of overweight including obesity among pupils at 6 years old was 32.11% respectively but at the age of 10, this prevalence was 27.51% with *p*-value of 0.007. Although the prevalence of overnutrition tended to decline for children from 6 to 10 years old, the prevalence was still very high.

This study also revealed a statistically significant difference in the prevalence of adverse nutritional status in the three socio-economic areas in Binh Dinh in which the prevalence of undernutrition is the highest in the mountainous area and midland. In contrast, the prevalence of overweight and obesity is the highest in the urban area. The prevalence of stunting among school-aged children was 12.5% in the mountainous area and midland and 2.34% in the urban area while the corresponding figures for overweight-obesity were 11.47% and 57.09%. This difference was also found in Ethiopia in which the prevalence of stunting was significantly lower in urban areas [33]. The prevalence of overweight and obesity in urban areas was relatively high which is, in fact, higher than that of primary schoolchildren in Ha Noi [15], Tien Hai [19] and Tay Ninh [26]. These results may be an important finding for managers in metropolitan areas. The prevalence of stunted pupils in the mountainous area and midland accounted for 12.5%. Stunting reflects a process of failure to reach linear growth potential as a result of suboptimal health and/or nutritional conditions. On a population basis, high levels of stunting are associated with poor socio-economic conditions and increase risk of frequent and early exposure to adverse conditions such as illnesses and/or inappropriate feeding practices. This prevalence was seriously high and 3 times higher than that of primary schoolchildren in the coastal plain districts of Binh Dinh Province (4.11%) with *p*-value of 0.00, but lower than that (28.6%) in Tien Hai-Thai Binh [19] and (18.7%) in Baghdad-Iraq [24]. The prevalence of underweight pupils was 23.14%, almost 3 times higher than that of primary school children in the coastal and plain districts of Binh Dinh Province (8.51%) and in Baghdad-Iraq (13.5%) [24] but the same as that in Tien Hai-Thai Binh (23.4%) [19]. Underweight might be secondary or symptomatic to an underlying disease; it is also may be severely deficient in nutrition and food. This form of malnutrition is a very common health problem in developing countries [34], especially in disadvantaged mountainous area and midland of Binh Dinh province. The prevalence of pupils with overweight-obesity was 11.47% in these areas and this prevalence was also rather high. The difference in prevalence of malnutrition forms among three study areas in Binh Dinh may be due to the differences in lifestyle and socio-economic status of these areas.

Trend analysis of adverse nutritional status by areas showed that the prevalence of overnutrition has decreased for children from 6 to 10 years old in the urban area but it was still very high whereas the prevalence of malnutrition forms was little changed over the age ranging from 6 to 10. The prevalence of undernutrition in mountainous area and midland was little changed over the ages; however, the prevalence of overweight and obesity tended to decline over the age groups. Thus, primary schoolchildren in Binh Dinh province are having two disadvantages in nutrition which are high prevalence of overweight-obesity in urban areas and gravely high prevalence of forms of undernutrition in mountainous area and midland. These are demonstrating the existence of the double burden of malnutrition among schoolchildren. The dual disadvantage of nutritional status of schoolchildren is also found in some studies in other countries [7],[35] and in Vietnam [17],[36]. These results suggest that it is important to explore potentially risky factors for overnutrition and undernutrition, including genetic factors and socio-economic status.

### Study limitations

Despite the potential contribution of the present study to the prevalence of malnutrition forms among primary schoolchildren in Binh Dinh province, there remain following limitations that are necessary to be mentioned. Firstly, the study did not assess the factors (dietary intake, family size, and mean income of caregivers, etc.) associated with malnutrition in this population, which could provide a further insight into the types of interventions to be implemented in study site. Secondly, this is a cross-sectional study and the temporal relationships between age groups and forms of malnutrition could not be established; however, we used the cross-sectional study as a surrogate for longitudinal one so further longitudinal studies are needed. Thirdly, because the number of pupils observed was very large, so the uncontrolled observational error may be a limitation of this study. Last but not least, we classified malnutrition by categorical variables without using the distribution of z scores for analysis, which probably ignored some the information available in this study.

## 5. Conclusion

It is undeniable that malnutrition forms among primary schoolchildren in Binh Dinh were high, especially the prevalence of thinness, stunting and underweight among pupils in mountainous area and midland were so high and did not seem to be improved over the ages. Moreover, the prevalence of overweight and obesity was relatively high in the urban areas. This has characterized an important public health challenge, highlighting the need for attention to potential interventions.
